# Increased surface expression of HIV-1 envelope is associated with improved antibody response in vaccinia prime/protein boost immunization

**DOI:** 10.1016/j.virol.2017.10.013

**Published:** 2018-01-15

**Authors:** Michael J. Hogan, Angela Conde-Motter, Andrea P.O. Jordan, Lifei Yang, Brad Cleveland, Wenjin Guo, Josephine Romano, Houping Ni, Norbert Pardi, Celia C. LaBranche, David C. Montefiori, Shiu-Lok Hu, James A. Hoxie, Drew Weissman

**Affiliations:** a Division of Infectious Diseases, Perelman School of Medicine, University of Pennsylvania, Philadelphia, PA, USA; b Division of Hematology and Oncology, Perelman School of Medicine, University of Pennsylvania, Philadelphia, PA, USA; c Department of Pharmaceutics, University of Washington, Seattle, WA, USA; d Department of Surgery, Duke University Medical Center, Durham, NC, USA; e Washington National Primate Research Center, University of Washington, Seattle, WA, USA

**Keywords:** CT, cytoplasmic tail, VACV, vaccinia virus, N7, N197Q, HIV, Envelope, Vaccine, Antibody, Vaccinia, Cytoplasmic tail

## Abstract

HIV-1 envelope (Env)-based vaccines have so far largely failed to induce antibodies that prevent HIV-1 infection. One factor proposed to limit the immunogenicity of cell-associated Env is its low level of expression on the cell surface, restricting accessibility to antibodies. Using a vaccinia prime/protein boost protocol in mice, we explored the immunologic effects of mutations in the Env cytoplasmic tail (CT) that increased surface expression, including partial truncation and ablation of a tyrosine-dependent endocytosis motif. After vaccinia primes, CT-modified Envs induced up to 7-fold higher gp120-specific IgG, and after gp120 protein boosts, they elicited up to 16-fold greater Tier-1 HIV-1 neutralizing antibody titers, although results were variable between isolates. These data indicate that the immunogenicity of HIV-1 Env in a prime/boost vaccine can be enhanced in a strain-dependent manner by CT mutations that increase Env surface expression, thus highlighting the importance of the prime in this vaccine format.

## Highlights

• Novel HIV Env cytoplasmic tail (CT) modifications increase surface expression.
• Vaccinia vector vaccination with CT-modified Envs induces high gp120-specific IgG.
• gp120 boosts in mice primed with CT-modified Envs induce high Tier-1 Nabs.

## 1 Introduction

The HIV-1 pandemic remains a major threat to global public health, with 2.6 million new infections annually, and a safe and effective vaccine is urgently needed (Wang et al., 2015; Harmon et al., 2016). Passive immunity experiments have demonstrated that anti-HIV-1 neutralizing antibodies (NAbs) can confer protection from infection in nonhuman primate models (Gautam et al., 2016, Parren et al., 2001, Hessell et al., 2009, Emini et al., 1992, Baba et al., 2000, Mascola et al., 2000); as a result, such antibodies are a major target of ongoing vaccine efforts (Haynes and Burton, 2017). The sole target of neutralizing or other protective antibodies on HIV-1 is the envelope glycoprotein (Env), which assembles as a trimer of heterodimers composed of surface gp120 and transmembrane gp41 subunits. The HIV-1 Env has evolved a variety of mechanisms to evade host antibody responses, including its ability to tolerate escape mutations in immunogenic epitopes (Wei et al., 2003, Moody et al., 2015), extensive glycosylation (Behrens et al., 2016, McCoy et al., 2016, Stewart-Jones et al., 2016, Zhou et al., 2017), conformational masking (Kwong et al., 2002), mimicry of host proteins (Yang et al., 2013), and low expression of Env on virus-infected cells and virions (Zhu et al., 2003, Chertova et al., 2002). Together, these features create substantial barriers to the design of an effective Env-based vaccine.

Despite extensive pre-clinical studies and six phase 2 or 3 clinical trials of HIV-1 vaccines (Rerks-Ngarm et al., 2009, The rgp120 HIV Vaccine Study Group, 2005, Buchbinder et al., 2008, Pitisuttithum et al., 2006, Gray et al., 2011, Hammer et al., 2013), only the RV144 trial has shown any efficacy, with 31.2% protection in humans at 42 months post-vaccination (Rerks-Ngarm et al., 2009). The RV144 vaccine regimen included four intramuscular inoculations of replication-incompetent canarypox vector expressing HIV-1 Gag, protease, and Env antigens and two injections of purified gp120 protein in alum. The primary correlates of protection were non-neutralizing IgG antibodies in plasma that bound to variable loops 1 and 2 (V1/V2) of gp120 and low levels of anti-Env IgA (Haynes et al., 2012). Secondary correlates in a post-hoc analysis included modest NAb activity and the ability of antibodies to mediate antibody-dependent cellular cytotoxicity (ADCC) on HIV-1-infected target cells (Haynes et al., 2012, Chung et al., 2014). These results have generated renewed interest in the potential advantages of poxvirus prime/protein boost vaccine approaches, as well as the antiviral functions of non-neutralizing antibodies (Corey et al., 2015, Ackerman et al., 2016, Huang et al., 2016, Forthal et al., 2013). Studies have since attempted to improve on the efficacy of RV144 by optimizing various aspects of the vaccine regimen, including dose schedule, vector, adjuvant, and Env sequence (Teigler et al., 2014; Vaccari et al., 2016; Easterhoff et al., 2017; NCT02404311, NCT02968849). There is considerable biological diversity among Env variants that can be used in the prime or boost, including differences in glycan organization (Stewart-Jones et al., 2016), epitope exposure (Sanders et al., 2013, De Taeye et al., 2015), and cell surface expression (Ye et al., 2004, Wyatt et al., 2008), but the exact contributions of these attributes to immunogenicity remain poorly understood.

The low expression of HIV-1 Env on the cell surface has long been hypothesized to impede an effective antibody response to membrane-associated forms of Env, as in viral infection or gene-based vaccines (Marsh et al., 1997). This low expression has been attributed at least in part to the presence of multiple endocytosis motifs within the long cytoplasmic tail (CT) of Env (Boge et al., 1998, Rowell et al., 1995, Bowers et al., 2000, Egan et al., 1996, Berlioz-torrent et al., 1999, Wyss et al., 2001, Byland et al., 2007). One well-described signal in the CT of HIV and SIV Envs is the membrane-proximal tyrosine (Tyr)-dependent endocytosis motif formed by the consensus amino acids GYxxΦ, where x is any amino acid and Φ is a bulky hydrophobic residue. This highly conserved motif binds to cellular adaptor protein complex 2 (AP-2) and recruits Env that is not incorporated into virions into clathrin-coated pits, thereby mediating internalization and clearance from the cell surface (Boge et al., 1998, Rowell et al., 1995, Bowers et al., 2000, Egan et al., 1996). GYxxΦ has also been shown to mediate directional budding of virus in polarized cell types (Lodge et al., 1997, Deschambeault et al., 1999) and to contribute to pathogenesis in SIV infection of pigtail macaques (Fultz et al., 2001, Breed et al., 2013, Breed et al., 2015). Additional but less well characterized internalization signals are present in the more distal CT (Wyss et al., 2001, Byland et al., 2007), consistent with the view that a low steady-state expression of Env on infected cells is an important and conserved viral property.

Surface expression of HIV and SIV Envs can be increased by mutations in the CT that ablate endocytosis signals. Our lab has previously described a variant of SIVmac251 termed CP-MAC that exhibited a marked increase in surface expression on infected cells. This increase was shown to be the result of a substitution of Tyr in the GYxxΦ motif and a premature stop codon immediately prior to the overlapping second exons of *tat* and *rev* (LaBranche et al., 1994, LaBranche et al., 1995, Sauter et al., 1996)—a truncation that arises commonly when SIVs are propagated in human cells (Kodama et al., 1989). It is unknown whether comparable mutations in the HIV-1 Env CT would confer a similar increase in surface expression, thereby making a potentially useful immunogen. At least two studies have directly compared the immunogenicity of HIV-1 Env CT mutants with increased surface expression to that of wild-type (WT) Envs (Ye et al., 2004, Wyatt et al., 2008), and both noted increases in gp120-binding IgG. However, the impact of high surface expression on IgG and especially NAb responses in the context of a viral prime/protein boost vaccine regimen has not yet been well defined.

In the present study, we used a clinically relevant vaccinia prime/gp120 protein boost protocol to evaluate the impact of mutations in the HIV-1 Env CT that ablate known endocytosis signals and increase expression on the cell surface. We hypothesized that the magnitude and particularly the quality of the antibody response would correlate with the level of Env cell surface expression driven by the vaccinia vector and thus the amount of native Env antigen available for interactions with B cells.

## 2 Materials and methods

### 2.1 Ethics statement

The investigators faithfully adhered to the “Guide for the Care and Use of Laboratory Animals” by the Committee for the Update of the Guide for the Care and Use of Laboratory Animals, Commission on Life Sciences, National Research Council. The animal facilities at the University of Pennsylvania are fully accredited by the American Association for Accreditation of Laboratory Animal Care (AAALAC). All studies were conducted under protocols approved by University of Pennsylvania IACUCs.

### 2.2 Generation of CT-modified HIV-1 Env constructs

**Fig. 1:**
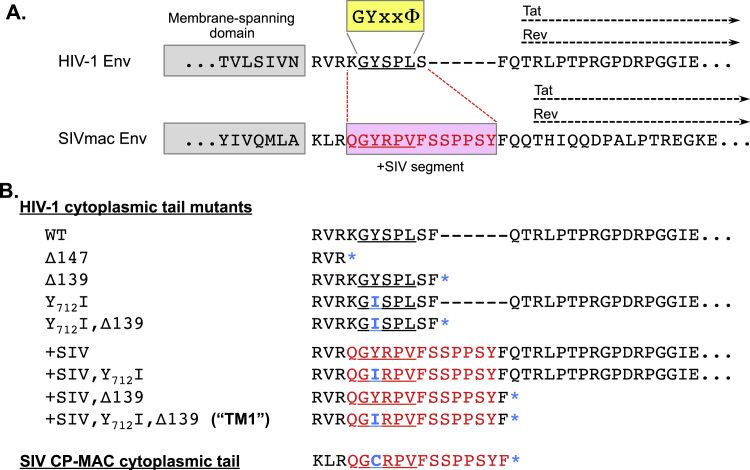
**Schematic of HIV-1 Env cytoplasmic tail mutants.** (A) Partial amino acid sequences of HIV-1 R3A and SIVmac239 Envs are shown, including part of the membrane-spanning domain and the highly conserved Tyr-dependent endocytosis motif (GYxxΦ). For both viruses, the positions overlapping the second exons of Tat and Rev in alternative reading frames are shown. The indicated segment from SIVmac (+SIV) was substituted into the HIV-1 Env CT to create Env constructs shown in Panel B. (B) HIV-1 Env CT mutants created to evaluate effects on Env surface expression. Substitutions included a Y_712_I substitution (HXB2 numbering) and/or a premature termination codon (*). Mutations were also made in the same positions in the Envs of HIV-1 89.6, 89.6 N7 (N_197_Q), and JRFL. Dashes (–) are used to facilitate alignment and highlight SIV residues with no homology in HIV-1 (SSPPSY). The sequence of SIV CP-MAC, which exhibits high levels of Env surface expression (LaBranche et al., 1994, LaBranche et al., 1995, Sauter et al., 1996), is shown for reference.

Mutant Env constructs were generated using HIV-1 R3A *env* (Meissner et al., 2004) (Genbank accession AY608577) in the pHSPG plasmid, HIV-1 89.6 *env* (Collman et al., 1992) (Genbank accession U39362) in the pCIneo plasmid (Promega), or JRFL *env* (Koyanagi et al., 1987) (Genbank accession U63632) in the pSVIII plasmid. The 89.6 and JRFL Envs each contain portions of sequence from strain HXB2 (Genbank accession K03455) based on their cloning strategies; these correspond to the signal peptide and the distal CT, C-terminal to amino acid 751 in HXB2 numbering. The 89.6 N7 (N_197_Q) mutant was generated from 89.6 WT, as previously described (Li et al., 2008). Amino acid sequences of all CT mutations used herein are shown in Fig. 1. Env CT mutant TM1 was generated in each Env isolate using a three-step process with QuikChange II XL reagents (Agilent) and the following primers: 1.) 5′-CAGAGTGCGGCAGGGCATCCGGCCAGTGAGCTTCTAGACACTGCTG-3′; 2.) 5′-GGCATCCGGCCAGTGTTCAGCTACTTCTAGACACTGCTG-3′; 3.) 5′-CGGCCAGTGTTCAGCAGCCCCCCCAGCTACTTCTAGACACTGCTG-3′.

### 2.3 Env expression analysis in plasmid-transfected HEK 293T cells

To measure the surface expression of CT-modified HIV-1 Envs, each Env mutant or WT/parental Env was co-transfected with pmax-GFP (Lonza) into human embryonic kidney (HEK) 293T cells (ATCC). HEK 293T cells were cultured in Dulbecco's modified Eagle medium (DMEM) supplemented with 2 mM l–glutamine (Life Technologies) and 10% fetal calf serum (FCS) (HyClone), and 9 μL of Fugene 6 was used for each transfection in a 12-well plate format. For 89.6 Envs in pCIneo vector, 1.5 μg Env plasmid and 0.3 μg GFP was used. For JRFL Envs in the lower-expressing pSVIII vector, a higher Env:GFP plasmid ratio was used: 1.5 μg Env and 0.1 μg GFP. The pHSPG vector expressing R3A Envs also expresses GFP, so 1.5 μg of pHSPG was used without separate GFP plasmid. At 18 h post-transfection, cells were harvested and stained for Env gp120 using 7.5 μg/mL 2G12 (obtained through the NIH AIDS Reagent Program from Polymun Scientific) and 1:300 secondary goat anti-human IgG Alexa Fluor 647 (Invitrogen A-21445). Cells were fixed with 4% paraformaldehyde and fluorescence was measured on a FACSCalibur (BD Biosciences). Analysis was performed using FlowJo software (Tree Star). Events were gated on GFP^+^ cells and median fluorescence intensity (MFI) was recorded and expressed relative to WT or parental Env.

### 2.4 Recombinant vaccinia viruses

HIV-1 Env-expressing recombinant vaccinia virus (VACV) vectors were made by cloning each *env* into a VACV shuttle vector, pGS20, under the control of the synthetic VACV early/late promoter, and then inserting it into the thymidine kinase gene of the v-NY strain of VACV (a replication-competent virus that was plaque purified from the New York City Board of Health strain) (Zarling et al., 1986, Cooney et al., 1993) by homologous recombination. Only 89.6 Envs in VACV vectors contain a C-terminal sequence from HXB2 (after amino acid 751 in HXB2), and both 89.6 and JRFL Envs contain the signal peptide sequence from HXB2. The negative control VACV vector for immunization studies was made similarly and encodes an irrelevant antigen, SIVmac239 Gag-Pol, instead of Env. The empty VACV vector used in vitro is the parental v-NY virus with an intact thymidine kinase gene.

Purified stocks, used for immunization studies, were prepared as follows: first, VACV-infected BSC40 cell pellets (~ 1 billion cells) were resuspended in 14 mL cold 10 mM Tris, pH = 9.0, transferred to a 40 mL glass Dounce homogenizer, and homogenized with 40 strokes of a tight pestle on ice. The material was centrifuged for 5 min at 1360 RPM at 4 °C, and supernatant was collected. 3 mL of cold 10 mM Tris was added and the material was centrifuged a second time. Supernatants from both spins were pooled and then sonicated in a 550 Sonic Dismembrator at an amplitude setting of 8. The material was sonicated at 1-min intervals three times in ice water with 1–3 min resting periods on ice. The material was gently layered onto 36% sucrose (10 mM Tris, pH = 9.0) and spun at 15,800 rpm in a Beckman Coulter SW 28 rotor for 80 min at 4 °C. The supernatant was aspirated and the virus pellet was resuspended in cold 1 mM Tris, pH = 9.0, and sonicated as above before being stored at −80 °C.

For in vitro Env expression studies, VACV crude lysates were prepared similarly as described above, but without sucrose gradient ultracentrifugation.

### 2.5 In vitro VACV infection and Env expression analysis

**Fig. 2:**
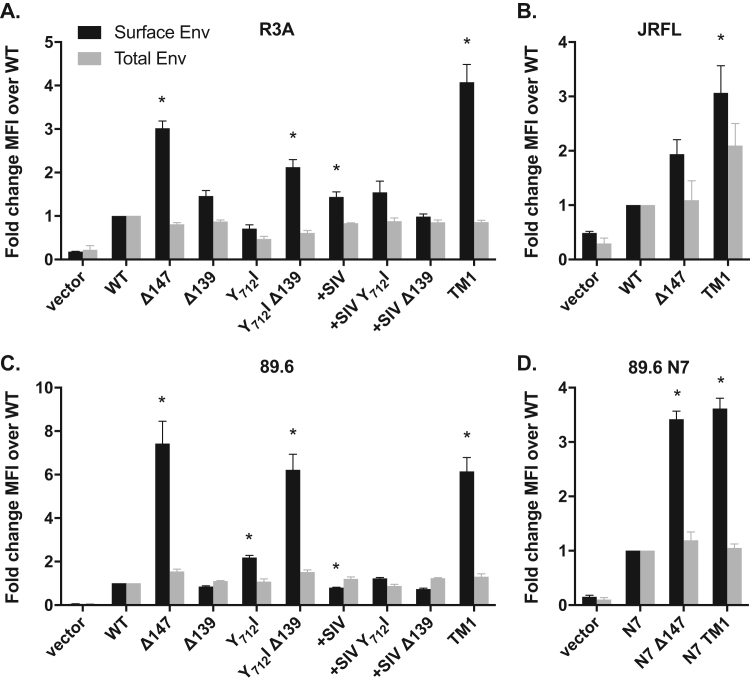
**CT mutations increase surface expression of Envs in plasmid-transfected HEK 293T cells.** The indicated CT mutations were made in four HIV-1 Envs: (A) R3A, (B) JRFL, (C) 89.6 WT, and (D) 89.6 N7 (N_197_Q). HEK 293T cells were co-transfected with plasmids encoding GFP and Env and at 18 h were stained for Env using mAb 2G12 under non-permeabilizing (surface stain) and permeabilizing (total stain) conditions. Data represent the average fold change in Env-specific mean fluorescence intensity (MFI) of GFP^+^ cells relative to parental Env in each experiment, with N = 3 (A, B, D) or N = 4 (C) replicates. Asterisks indicate Env variants that showed a significant fold difference (p < 0.05) in surface expression compared to the parent Env (normalized to 1) by one-sample *t*-test on log-transformed data with Bonferroni correction.

**Fig. 3:**
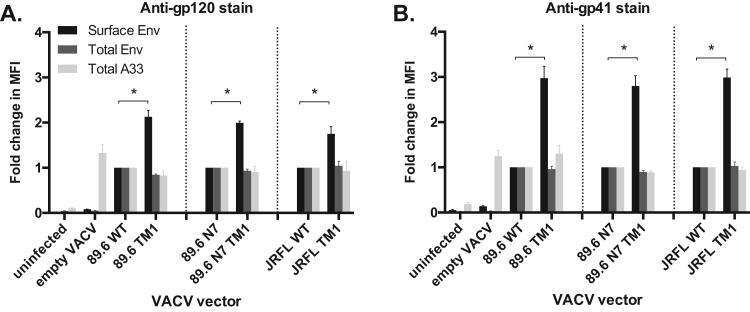
**CT mutations increase surface expression of Envs in recombinant VACV-infected cells**. HEK 293T cells were infected (MOI = 3) with the indicated Env-expressing recombinant VACV or parental VACV (empty). After 16 h, cells were stained for Env using (A) 2G12, an anti-gp120 mAb, or (B) 2F5, an anti-gp41 mAb under permeabilizing (total) or non-permeabilizing (surface) conditions (N = 3). In each experiment, VACV A33 protein staining was performed under permeabilizing conditions to control for transduction efficiency and to gate on infected cells. The MFI of Env and A33 stains are shown relative to parental Envs, with comparison groups separated by dotted lines. Asterisks indicate a significant fold difference (p < 0.05) in surface expression of WT and TM1 Envs by one-sample *t*-test on log-transformed data with Bonferroni correction.

For analyses of Env expressed by VACV vectors, 1.6 × 10^6^ HEK 293T cells were infected with VACV crude lysates at a multiplicity of infection (MOI) of 3. Virus was absorbed onto cells at 37 °C for 1 h in 0.5 mL DPBS with 10 mM MgCl_2_ and 0.01% BSA, and then replaced with 2 mL of DMEM with 10% FBS. The infection was allowed to proceed for 16 h post-absorption. Harvested cells were stained for HIV-1 Env with human mAbs 2G12 or 2F5 (NIH AIDS Reagent Program) at 7.5 μg/mL and 10 μg/mL, respectively, and 1:100 secondary goat anti-human IgG Alexa Fluor 647 conjugate (Invitrogen A-21445). VACV A33 protein was stained using 1:300 rabbit anti-A33 serum (kindly provided by Dr. Stuart Isaacs) and 7.5 μg/mL goat anti-rabbit IgG FITC conjugate (BD Biosciences 552420). Each antibody was incubated on cells for 30 min on ice, and each sample was stained with and without permeabilization with 0.1% saponin to allow total cellular stain. All samples were fixed with paraformaldehyde after staining and fluorescence was measured using a FACSCalibur flow cytometer. Ten thousand events were collected and live, A33^+^ cells were gated on to determine the mean fluorescence intensity (MFI) of Env and A33 staining (Fig. 2, Fig. 3).

**Fig. 4:**
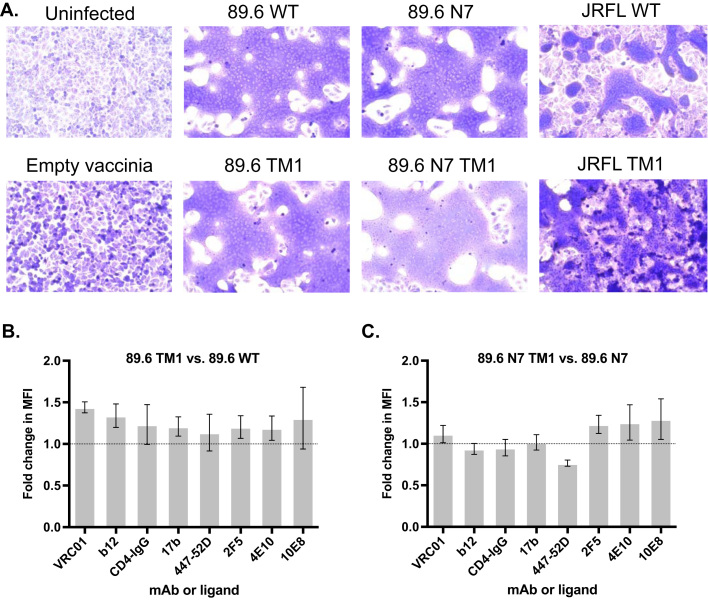
**TM1 modified Env is functional and maintains broadly neutralizing antibody epitopes.** (A) A cell-cell fusion assay was performed by infecting (MOI = 1) HIV-permissive TZM-bl cells with recombinant VACV vectors encoding HIV-1 Envs with WT or TM1 mutated CTs or a control VACV encoding no recombinant gene. Cells were fixed at 18 h and syncytia were visualized by Giemsa stain. (B, C) HEK 293T cells were transduced with recombinant VACV vectors encoding (B) the HIV-1 89.6 or (C) 89.6 N7 (N_197_Q) Envs with WT or TM1 mutated CTs, and cells were stained (N = 3) under non-permeabilizing conditions with the indicated mAbs or a CD4-IgG fusion protein. Fold changes in MFI (TM1:WT) for each mAb were normalized to the fold change in 2G12 stain to account for differences in surface expression. No significant fold differences (p > 0.05) relative to parental Env (dashed line) were noted using one-sample *t*-test on log-transformed data with Bonferroni correction.

For antigenicity analysis, HEK 293T cells were infected as above and stained without permeabilization using 1:300 rabbit anti-A33 serum and the Env-specific mAbs 2G12, VRC01, b12, CD4-IgG,17b, 447-52D, PG9, PG16, 2F5, 4E10, 10E8 (NIH AIDS Reagent Program), which bind to different antigenic determinants. Goat anti-rabbit IgG FITC and goat anti-human IgG Alexa Fluor 647 conjugates were used as secondary antibodies. Transduced cells were gated on using the A33 stain to determine the MFI for each mAb. The ratio of the MFI of TM1 variants relative to their parental counterparts was calculated for each mAb, and each of these raw fold differences was divided by that of the 2G12 stain, as previously done (Veillette et al., 2014), to eliminate fold differences due to surface expression levels. These 2G12-normalized fold differences are shown in Fig. 4B and C.

### 2.6 Cell-cell fusion assay

The functionality of CT-modified Envs was qualitatively assessed using a cell-cell fusion assay, as described previously (Bolmstedt et al., 1991). TZM-bl cells (provided by Dr. John Kappes, Dr. Xiaoyun Wu, and Tranzyme Inc. through the NIH AIDS Reagent Program) were infected with crude lysates of recombinant VACV vectors at an MOI of 1.0. After 18 h of infection, cells were fixed in a solution of 95% ethanol and 5% acetic acid and stained with Giemsa to visualize syncytia.

### 2.7 gp120 expression and purification

89.6 N7 gp120 and JRFL WT gp120 were expressed in BSC40 cells by recombinant VACV infection and purified by a three-step procedure using lectin affinity, diethylaminoethanol (DEAE) anion exchange, and size exclusion chromatography, as previously described (Guo et al., 2013).

### 2.8 Mice

Female C57BL/6 (BL/6) mice were purchased from NCI and housed in a BSL2 containment facility. All mice were 6 weeks of age at the initiation of immunization studies.

### 2.9 Immunizations

Mice were immunized intraperitoneally with 1 × 10^8^ pfu of recombinant VACV (v-NY strain) encoding HIV-1 Env variants or SIV Gag-Pol at weeks 0 and 4. At weeks 8 and 12, mice were immunized intramuscularly with 5 μg gp120 in PBS in 1% Alhydrogel alum adjuvant (Invivogen). Serum samples were collected prior to first immunization and immediately prior to each subsequent prime or boost. Splenectomies and final serum collection were performed two weeks after the second gp120 boost (week 14).

### 2.10 Enzyme-linked immunosorbent assays (ELISAs)

HIV-1 gp120-specific and gp41-specific IgG in mouse serum was quantified by ELISA. Immulon 4 HBX high-binding plates were coated with purified HIV-1 89.6 N7 or JRFL WT gp120 protein, or with MN gp41 protein containing truncations of the fusion peptide, membrane-spanning domain, and distal portion of CT (provided by ImmunoDx through NIH AIDS Reagent Program), all at a final concentration of 1 µg/mL in PBS overnight at 4 °C. Subsequent incubation steps were performed at RT in 100  μL volumes. Plates were washed once with wash buffer (0.05% Tween-20 in PBS) and blocked with blocking buffer (2% BSA in PBS) for 1 h, followed by three more washes. Dilutions of sera and standard were made in blocking buffer and incubated on plates for 1.5 h. Murine mAbs against gp120 (3B3, obtained from the Duke Human Vaccine Institute, NIH AIDS Reagent Program) and gp41 (D50, provided by Dr. Patricia Earl, NIAID, NIH AIDS Reagent Program) were used as standards to estimate the concentration of specific IgG in µg/mL. After incubation, samples were removed and plates were washed four times with wash buffer. Goat anti-mouse IgG HRP conjugate (Sigma-Aldrich A8924) at 1:10,000 in blocking buffer was incubated for 1 h. After four washes, TMB substrate mixture (KPL) was added at 100 µL/well for 20  min. 50 µL per well of 2 N sulfuric acid was used to stop the reaction, and the optical density (OD) was read at 450 nm on a Dynex MRX Revelation microplate reader.

VACV-, V3-, and V1/V2-specific IgG ELISAs were performed similarly to gp120-specific IgG ELISA, with the following modifications. For VACV, the coating antigen was Western Reserve VACV lysed in RIPA buffer, diluted 1:200 in PBS (kindly provided by Dr. Stuart Isaacs). For V3, the coating antigen was synthetic 89.6 Env V3 peptide (GenScript) at 5 μg/mL in PBS. For V1/V2, the coating antigen was a scaffold protein containing HIV-1 92US715 V1/V2 fused to MLV gp70 (kindly provided by Dr. Shan Lu) at 1 μg/mL in PBS.

### 2.11 Splenocyte stimulation and intracellular cytokine staining

To perform Env-specific and VACV-specific T cell analyses, splenocytes (2 × 10^6^) were incubated with four separate SHIV 89.6P Env peptide pools (NIH AIDS Reagent Program) and one immunodominant VACV peptide pool (NR-4058, BEI Resources) at 37 °C and 5% CO_2_. All peptides were used at 1 µg/mL, and DMSO was used as a control for background. After 1 h, GolgiPlug (brefeldin A), GolgiStop (monensin), and anti-CD107a-FITC (BD Biosciences) were added and cells were incubated for an additional 5 h. Cells were washed in PBS and resuspended in LIVE/DEAD Aqua Blue stain (Invitrogen) for 10 min at room temperature (RT). A mixture of CD44-PE/Cy5, CD27-PE (BD Biosciences), CD8-Pacific Blue, and CXCR5-Brilliant Violet 605 (BioLegend) surface marker antibodies was added and incubated for 30 min at RT. Cells were washed in FACS buffer (PBS, 1% FBS), resuspended in FIX & PERM (BD Biosciences), and incubated for 20 min at RT. Cells were washed with Perm/Wash (BD Biosciences) and then resuspended in a mixture of TNFα-PE/Cy7, IFNγ-Alexa Fluor 700, IL2-APC, and CD3-APC/Cy7 antibodies (BD Biosciences) and incubated at RT for 1 h. Cells were washed, resuspended in 1% paraformaldehyde in PBS, and analyzed for fluorescence using an LSRII (BD Biosciences). Analysis was performed using FlowJo software (Tree Star). Events were gated on live (Aqua Blue-negative) cells. CD4^+^ cells were identified as CD3^+^/CD8^-^ cells. Background-subtracted percentages of cytokine-positive cells in each of four Env peptide pools were added together to yield the Env-specific CD4^+^ T cell response.

### 2.12 Neutralization assays

Pseudotype virus neutralization assays were performed in TZM-bl reporter cells, as previously described (Montefiori, 2005) using pre-immune and post-immune mouse sera. Samples were assayed for neutralization of pseudoviruses expressing multiple HIV-1 Envs or murine leukemia virus Env to measure nonspecific neutralization and/or cytotoxicity. Values were reported as the reciprocal of the serum dilution at which 50% reduction of luciferase activity is observed (ID_50_).

### 2.13 Statistical analysis

Statistical tests were performed using GraphPad Prism software. IgG concentrations and data expressed as fold changes were log-transformed before analysis. Normally distributed data were analyzed by one-way ANOVA with Tukey test or one-sample *t*-test with Bonferroni correction. Non-parametric data were analyzed by the Mann-Whitney test or Kruskal-Wallis test with Dunn's correction for multiple comparisons.

## 3 Results

### 3.1 Cytoplasmic tail (CT) mutations can increase HIV-1 Env surface expression in transfected HEK 293T cells

We introduced several mutations into the CT of the clade B HIV-1 R3A Env (Meissner et al., 2004) that were designed to alter cell surface expression. Fig. 1A shows the organization of the HIV-1 CT and Fig. 1B shows mutations that were introduced, including combinations of: (1) a premature stop codon that removed the distal 147 of 151 predicted amino acids in the CT, removing all known trafficking motifs (denoted as ∆147) (Yue et al., 2009); (2) a premature stop codon that removed 139 amino acids, leaving intact the membrane-proximal endocytosis signal, ^711^GYSPL^715^ (denoted as Δ139); and (3) a point mutation, Y_712_I, that ablated this motif. Interestingly, we noted that the SIVmac Env CT contains six amino acids (SSPPSY) prior to the start of the second exons of *tat* and *rev* that have no homology in HIV-1 (Fig. 1A) (Santos da Silva et al., 2013). SIV Env mutants with high surface expression, such as CP-MAC (LaBranche et al., 1995), often have premature stop codons between these six residues and the *tat* and *rev* overlapping reading frames (Kodama et al., 1989, Hirsch et al., 1989). Considering that these residues could contribute to a trafficking motif regulating Env surface expression, we introduced a 13-amino acid segment spanning this region of the SIV Env CT into HIV-1 (denoted as +SIV). On this chimeric background, we then introduced Y_712_I, ∆139, or both changes together. For simplicity, the latter construct is referred to as TM1 (tail modification 1). A similar set of mutations was also made in the Envs from clade B HIV-1 strains 89.6 and JRFL.

The effects of CT mutations on surface expression were first evaluated in the context of HIV-1 R3A Env. Plasmids encoding R3A Env CT variants were transfected into HEK 293T cells, and surface and total expression were measured by flow cytometry (Fig. 2A). Three Env mutants exhibited ≥ 2-fold increases in surface expression relative to WT: Y_712_I Δ139 (2-fold, p = 0.09), Δ147 (3-fold, p = 0.02), and TM1 (4-fold, p = 0.04). All Envs exhibited similar total cellular expression levels when assays were performed on permeabilized cells.

We next extended this evaluation to assess the effects of analogous mutations on 89.6 and JRFL Envs (Fig. 2B–D). In 89.6 Env, the CT mutations producing the highest surface expression were similarly Y_712_I Δ139, Δ147, and TM1 6–7-fold, p < 0.01). CT mutations were also studied in the context of the 89.6 N7 Env variant, which contains a deletion of a single glycosylation site (N_197_Q) (Townsley et al., 2015, Liang et al., 2016) near the CD4 binding site and second variable loop (V2). Previously, it was found that the N_197_Q mutation in 89.6 Env markedly increased NAb responses against autologous and heterologous viruses in a vaccinia prime/gp120 protein boost immunization of pigtail macaques (Li et al., 2008, Townsley et al., 2016). In 89.6 N7 Env, the Δ147 and TM1 mutations generated 3–4-fold (p < 0.01) increases in Env surface expression. A similar increase in surface Env was also observed for the JRFL Env TM1 mutant (3-fold, p = 0.04). Thus, across several HIV-1 Envs, CT mutations conferred an increase in surface expression, particularly constructs with the TM1 mutations (i.e. the +SIV segment, Y_712_I, and ∆139), and this variant was selected for further study as an immunogen using a vaccinia expression protocol.

### 3.2 CT mutations increase Env surface expression in a vaccinia virus expression system

**Fig S1:**
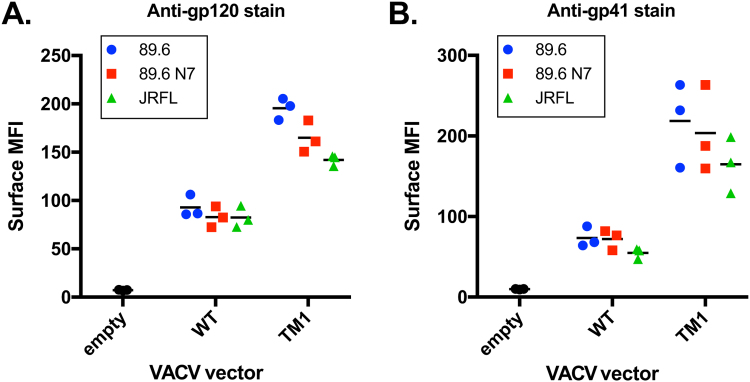
**Similar WT and TM1 surface expression among multiple strains of Env expressed by recombinant VACV.** In the same experiments shown in Fig. 3, HEK 293T cells were infected (MOI = 3) recombinant VACV and stained for VACV A33 to gate on infected cells and for Env using (A) 2G12, an anti-gp120 mAb, or (B) 2F5, an anti-gp41 mAb under non-permeabilizing conditions (n = 3 independent experiments). The un-normalized mean fluorescence intensity (MFI) of the Env stains is shown. Statistical analysis is described in Fig. 3.

89.6, 89.6 N7, and JRFL Envs with unmutated or TM1-modified CTs were inserted into vaccinia virus (VACV) vectors derived from the replication-competent v-NY strain (Zarling et al., 1986, Cooney et al., 1993). HEK 293T cells were infected with parental VACV or recombinant VACV vectors expressing Envs with or without the TM1 mutations. Surface and total Env expression were assessed by flow cytometry using antibodies 2G12 and 2F5, which bind to gp120 and gp41, respectively (Fig. 3 and Supp. Fig. 1). TM1-modified 89.6, 89.6 N7, and JRFL Envs exhibited a roughly 2-fold increase in gp120 and a 3-fold increase in gp41 surface expression (all p < 0.05), with no differences in total cellular Env (Fig. 3). To control for transduction efficiency, VACV A33 protein expression was also assessed, and no significant differences were detected. Thus, similar to their expression from plasmid in HEK 293T cells, HIV-1 Envs containing the TM1 mutations also exhibited increased cell surface expression using a VACV expression system, making them amenable to a comparative immunogenicity study using a VACV prime/protein boost vaccine regimen.

### 3.3 Envs containing CT mutations for high surface expression mediate fusion and maintain epitopes for broadly neutralizing antibodies

We sought to determine whether the TM1 CT mutations altered Env function or the exposure of epitopes to which broadly neutralizing antibodies are directed. CT truncations in HIV-1 Env have previously been shown to affect fusion efficiency and antigenicity of the ectodomain (Yue et al., 2009, Edwards et al., 2002, Chen et al., 2015, Wyss et al., 2005). In a cell-cell fusion assay, VACV-expressed TM1 Envs induced syncytium formation similarly to parental 89.6, 89.6 N7, and JRFL Envs, indicating that TM1-modified Envs maintain fusogenic function (Fig. 4A). Next, Envs with or without the TM1 mutations were expressed in HEK 293T cells and analyzed by flow cytometry for the relative expression of antibody epitopes including the CD4 binding site (VRC01, b12, CD4-IgG), co-receptor binding site (17b), V3 loop crown (447-52D), and the membrane-proximal external region (MPER) in gp41 (2F5, 4E10, 10E8) (Fig. 4B and C). No significant differences in antibody/ligand binding were observed between WT and TM1 Envs, indicating that TM1 modification did not result in major antigenic changes within the epitopes for several groups of broadly neutralizing antibodies.

### 3.4 Immunization with CT-modified Envs in a vaccinia prime/protein boost protocol generates higher levels of gp120-specific IgG

The immunogenicity of Envs containing the TM1 mutations was evaluated using a poxvirus prime/protein boost strategy, analogous to the RV144 regimen that showed partial protective efficacy in humans (Rerks-Ngarm et al., 2009). Our regimen used Env-expressing recombinant VACV vectors for the primes followed by purified gp120 protein for the boosts. We hypothesized that the level of Env surface expression driven by the priming vectors would correlate with the magnitude and quality of Env-specific antibody responses after the primes and/or the boosts.

**Fig. 5:**
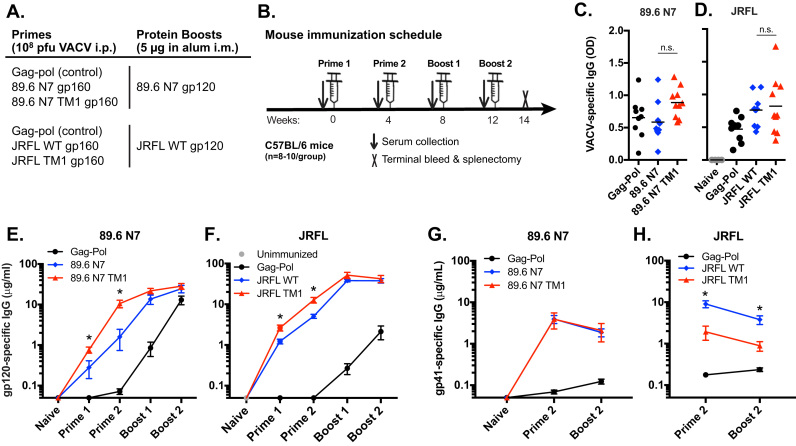
**Increased gp120-specific IgG responses in mice primed with TM1 modified Envs.** (A) Immunogens and doses used in prime/boost immunizations. (B) Timeline of immunizations and sample collection. (C, D) VACV lysate-specific IgG was measured by ELISA using serum from mice primed twice with VACV vectors encoding (C) 89.6 N7 or (D) JRFL Envs or Gag-Pol. Data represent the mean OD using serum diluted (C) 1:20,000 and (D) 1:30,000. (E) 89.6 N7 gp120-specific IgG and (F) JRFL gp120-specific IgG were measured by ELISA in mice primed with the indicated VACV vectors and boosted with (E) 89.6 N7 gp120 or (F) JRFL gp120. (G, H) MN gp41-specific IgG was measured by ELISA for both (G) 89.6 N7 and (H) JRFL immunizations. Time points on graphs correspond to the sera collected after the indicated immunizations. N = 8–10 mice per immunized group. Separate unimmunized mice (N = 4) were used as negative controls in (D) and (F). Error bars indicate the SEM, and asterisks denote a significant difference (p < 0.05) between WT CT and TM1 groups by one-way ANOVA with Tukey's test on log-transformed data.

The 89.6 N7 and JRFL Envs were selected for evaluation in immunization studies based on previous work demonstrating the potential of 89.6 N7 to generate potent and broad NAb responses (Li et al., 2008) and due to the abundance of structural and immunologic information available for JRFL (Crooks et al., 2015, Sharma et al., 2015, Feng et al., 2016, Guenaga et al., 2016, Munro et al., 2014). C57BL/6 mice (N = 8–10/group) were injected intraperitoneally (i.p.) at weeks 0 and 4 with 10^8^ pfu of VACV encoding 89.6 N7 or JRFL Envs with WT or TM1-modified CTs (Fig. 5A and B). A VACV vector encoding SIV Gag-Pol and lacking Env was used as a control. All groups of mice, including VACV Gag-Pol, were boosted at weeks 8 and 12 with intramuscular (i.m.) injections of 5 μg of 89.6 N7 or JRFL WT gp120 protein in 1% alum.

**Fig S2:**
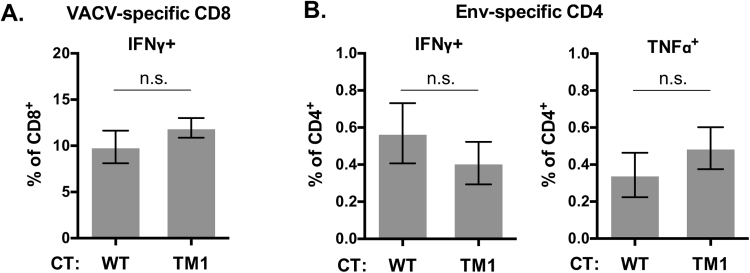
**No significant difference in VACV-specific CD8**^**+**^**or Env-specific CD4**^**+**^**T cell responses resulting from TM1 CT modification.** Splenocytes from mice immunized with 89.6 N7 Envs with WT or TM1 CT were stimulated with peptide pools from (A) VACV or (B) SHIV 89.6P Env and were stained for intracellular cytokine expression. Data represent mean frequencies of IFNγ^+^ and TNFα^+^ cells, as a percentage of total CD8^+^ or CD4^+^ T cells, and error bars are the SEM; n.s. = not significant (p > 0.05) by Mann-Whitney test.

To determine if mice received comparable effective doses of vector, VACV-specific antibody responses were measured by ELISA. In both immunization series, there were no significant differences between groups in VACV lysate-specific IgG measured 4 weeks after the second VACV prime (Fig. 5C and D). Consistent with this result, we also observed statistically equivalent VACV-specific CD8^+^ T cell (Supp. Fig. 2A) and Env-specific CD4^+^ T cell responses (Supp. Fig. 2B) elicited by vectors expressing 89.6 N7 Env with or without the TM1 mutations at the time of euthanasia, two weeks after the second boost. The VACV-specific CD4^+^ T cell and Env-specific CD8^+^ T cell responses were negligible with the peptide pools used (data not shown).

**Fig S3:**
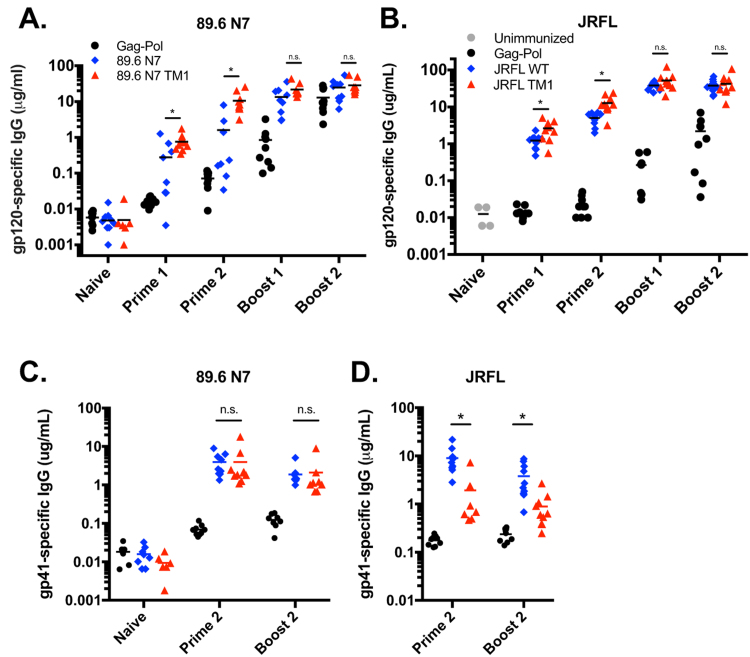
**Distribution of gp120- and gp41-specific IgG responses.** The data reflect the same assays shown in Fig. 5 but gp120-specific IgG (A, B) and gp41-specific IgG (C, D) is shown for individual mice in (A, C) 89.6 N7 and (B,D) JRFL immunizations. Horizontal bars indicate the mean. Separate unimmunized mice were used as negative controls in (B). Asterisks denote a significant difference (p < 0.05) between WT CT and TM1 groups by one-way ANOVA with Tukey's test on log-transformed data.

Gp120-specific IgG responses were measured by ELISA after each immunization as outlined in Fig. 5B. Mice that were primed with 89.6 N7 TM1 Env generated significantly higher gp120-binding IgG than those primed with 89.6 N7 (Fig. 5E and Supp. Fig. 3A), with a 3-fold increase after the first prime and a 7-fold increase after the second prime. A similar effect was noted in the JRFL immunization, although to a lesser extent: mice primed with JRFL TM1 Env developed 2-fold higher gp120-specific IgG after one or two primes compared to JRFL WT (Fig. 5F and Supp. Fig. 3B). In both immunization series, the gp120 protein boosts increased the levels of gp120-specific IgG while reducing the fold difference between the parental and TM1 Env groups. Interestingly, in mice primed with the Gag-Pol VACV vector, boosting with 89.6 N7 gp120 appeared more effective than JRFL gp120. The mechanism of this effect is unclear but appears to be intrinsic to the Env strain, since the gp120 proteins were similarly prepared and had comparable purity.

Surprisingly, the gp41-specific IgG response did not follow the same trends as the gp120-specific IgG. Mice primed with 89.6 N7 TM1 generated equivalent gp41-specific IgG compared to mice primed with parental 89.6 N7 (Fig. 5G and Supp. Fig. 3C). In contrast, mice primed with JRFL TM1 mounted a 5-fold lower gp41-specific response than mice receiving JRFL WT (Fig. 5H and Supp. Fig. 3D). In both immunizations, the gp41-specific IgG waned slightly when sampled after the gp120 boosts, as expected. The effect of TM1 modification on gp41-specific IgG was thus variable depending on the Env isolate.

### 3.5 Prime/boost immunization with CT-modified Envs generates higher HIV-1 NAb titers

**Fig. 6:**
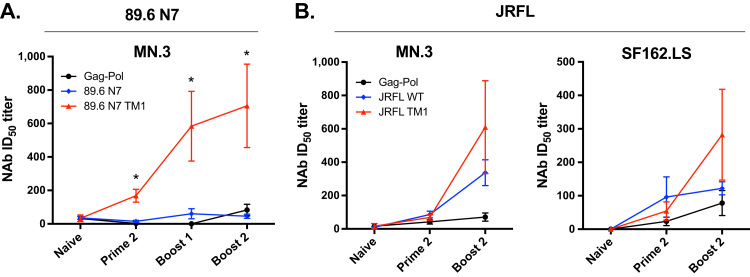
**Increase in HIV-1 NAb responses in mice primed with TM1 modified Envs.** NAb titers against HIV-1 MN.3 and SF162.LS pseudoviruses at the indicated time points were measured using TZM-bl reporter cells. The mean ID_50_ titers are shown for (A) 89.6 N7 and (B) JRFL Env immunization series. N = 8–10 mice per immunized group. Background activity against a murine leukemia virus Env pseudovirus is subtracted from all data except the naive time point in (A), for which serum volume was limited. Error bars indicate the SEM, and asterisks denote a significant difference (p < 0.05) in NAb titer between WT and TM1 CT groups by Kruskal-Wallis test with Dunn's correction.

**Fig S4:**
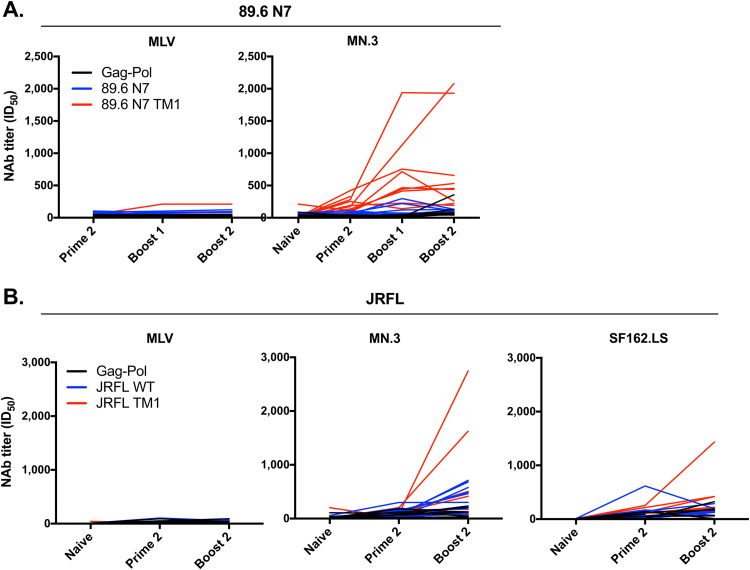
**Distribution of HIV-1 NAb responses and low non-specific activity.** The data reflect the same assays as Fig. 6 but are shown for individual animals without subtracting non-specific neutralizing activity quantified using a murine leukemia virus (MLV) Env pseudovirus. NAb titers are shown for MLV and HIV-1 strains MN.3 and SF162.LS for 8–10 mice per group in (A) 89.6 N7 and (B) JRFL Env immunizations. Statistical analysis of background-corrected data is shown in Fig. 6.

We next determined whether increased surface expression of Env could impact the development of anti-HIV-1 NAbs. Using a TZM-bl pseudovirus reporter assay, we found that mice primed with 89.6 N7 Env containing the TM1 mutations generated markedly higher NAb titers compared to mice primed with parental 89.6 N7 (Fig. 6A and Supp. Fig. 4A). Neutralization of the Tier-1A (highly neutralization-sensitive) strain MN.3 was increased 11-fold after the second prime (p = 0.02), 10-fold after the first boost (p = 0.02), and 16-fold after second boost (p = 0.0007) (Fig. 6A). These findings were striking, given that there was no significant difference in gp120-specific or gp41-specific IgG between 89.6 N7 and 89.6 N7 TM1 vaccinations after protein boosts (Fig. 5E and F). The breadth of the Tier-1 NAb activity in mice immunized with 89.6 N7 TM1 is not known, as serum was not available to measure neutralization of other Tier-1 strains.

In contrast to the findings for 89.6 N7 Envs, mice vaccinated with JRFL Envs with or without the TM1 mutations generated low NAb titers to Tier-1A MN.3 and SF162.LS strains after two VACV primes. After two protein boosts, mice immunized with JRFL TM1 Env exhibited a 2-fold higher mean NAb titer against these strains, although responses were highly variable, and the difference was not statistically significant (Fig. 6B and Supp. Fig. 4B). No consistent NAb activity was elicited by JRFL WT or TM1 against the moderately neutralization-sensitive Tier-1B strain BaL.26 (data not shown). In both JRFL and 89.6 N7 immunizations, no significant NAb activity could be determined against more neutralization-resistant (Tier-2) viruses, including X2278 and autologous JRFL or 89.6, when measured at the terminal time point (data not shown).

Thus, we observed similar overall trends for 89.6 N7 and JRFL Env immunizations, in which Envs modified for increased surface expression generated enhanced levels of gp120-specific IgG and Tier-1 NAbs. These effects were greatest with the 89.6 N7 TM1 Env, where pronounced increases in anti-gp120 IgG and NAb were observed after VACV primes and even greater increases in NAbs were noted after gp120 boosts.

### 3.6 CT modification does not change the immunogenicity of variable loops 1/2 and 3

**Fig S5:**
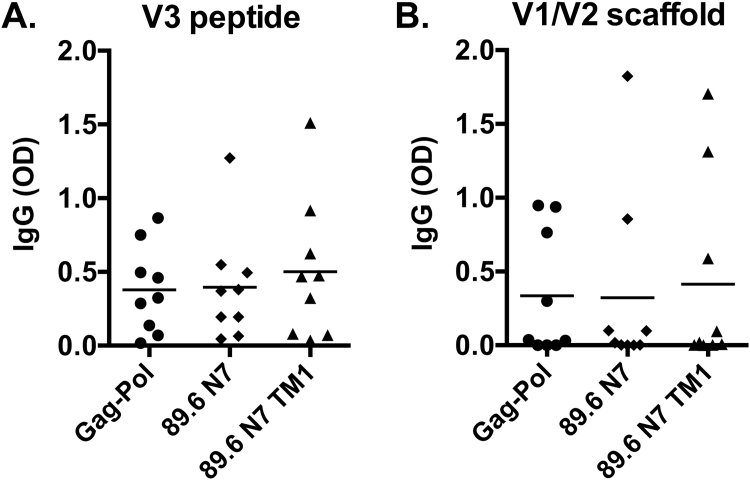
**No difference in V3 and V1/V2 epitope-specific IgG responses due to TM1 modification.** (A) V3-specific and (B) V1/V2-specific IgG responses were assayed by ELISA using synthetic V3 peptide and MLV gp70-scaffolded V1/V2, respectively, as the coating antigens and post-boost two serum diluted (A) 1:2000 or (B) 1:200. Points represent OD values for individual mice and horizontal lines indicate the mean. Groups were not statistically different (p > 0.05) by Kruskal-Wallis test with Dunn's correction.

NAb responses to Tier-1 HIV-1 isolates have previously been attributed to antibodies binding to hypervariable loops on gp120, particularly the V3 loop (Zolla-pazner et al., 2016, Hioe et al., 2010). We investigated this possibility by evaluating sera from mice immunized with 89.6 N7 and 89.6 N7 TM1 Envs for binding to a V3 peptide from 89.6 Env. As shown in Supp. Fig. 5A, no difference in this response was observed. In addition, epitopes within the V1/V2 loops have been shown to be highly immunogenic and a target for neutralizing and non-neutralizing antibodies in various vaccine regimens (Haynes et al., 2012, Rolland et al., 2012, Liao et al., 2013). However, when sera from mice immunized with 89.6 N7 Envs were assessed for IgG to V1/V2 presented on a murine leukemia virus gp70 scaffold (Haynes et al., 2012, Zolla-Pazner et al., 2014), only ~ 50% of mice made detectable V1/V2-specific IgG responses, and there was no difference between mice receiving parental or TM1-modified 89.6 N7 Envs (Supp. Fig. 5B). Thus, although immunodominant in several vaccine protocols, antibody responses to V3 and V1/V2 loops were unaffected by differences in Env cell surface expression in this VACV prime/gp120 boost protocol.

## 4 Discussion

HIV and SIV Env CTs contain a membrane-proximal Tyr-dependent endocytosis signal that serves to reduce the steady-state expression of Env on infected cells (Boge et al., 1998, Rowell et al., 1995, Bowers et al., 2000, Egan et al., 1996). The finding that this motif is absolutely conserved among the majority of primate and non-primate lentiviruses (Santos da Silva et al., 2013) and that there are additional but less well characterized endocytic motifs in more distal regions of these tails (Rowell et al., 1995, Bowers et al., 2000, Byland et al., 2007) has suggested that endocytic trafficking of Env could play an important role in pathogenesis, possibly by protecting virus-producing cells from humoral immune responses such as ADCC (von Bredow et al., 2015). Indeed, for the pathogenic SIV molecular clone, SIVmac239, ablation of the membrane-proximal endocytosis signal (GYRPV) by deletion of GY_721–722_ results in a virus that is highly replication-competent in vivo but is susceptible to host immune control in pigtail macaques (Fultz et al., 2001, Breed et al., 2013, Breed et al., 2015). The aim of the current study was to determine if mutations in the HIV-1 Env CT that ablated endocytosis motifs and, as a result, increased cell surface expression could impact Env immunogenicity in a VACV prime/protein boost vaccine regimen. Given recent findings in the RV144 vaccine trial that a poxvirus prime/protein boost protocol could confer partial protection from HIV infection and that this effect correlated with antibody responses to gp120 (Haynes et al., 2012, Chung et al., 2014), we reasoned that modifications to Env that increased its expression on the cell surface could be a useful adjunct to improve the efficacy of this approach, particularly with regard to the poxvirus prime in which Env can be expressed as a membrane-associated trimer on antigen-presenting cells.

We evaluated several HIV-1 Env CT modifications that were informed by our prior studies of CP-MAC, an in vitro derived variant of SIVmac251 that exhibited a marked increase in Env surface expression on infected cells (LaBranche et al., 1994, LaBranche et al., 1995). We showed previously that this phenotype resulted from the combined effects of a premature stop codon in the CT and loss of the membrane-proximal, Tyr-dependent endocytosis motif. In the current study, we evaluated similar mutations (Y_712_I and ∆139) in the HIV-1 Env CT and, based on differences in the organization of the SIV and HIV-1 CTs, created a set of novel HIV-1 Env chimeras containing a segment (+SIV) of the SIVmac CT spanning from the Tyr motif to the site of the stop codon in CP-MAC (Fig. 1). The chimera containing all of these changes, termed TM1, exhibited a marked increase in surface expression compared to Envs with unmutated CT, ranging from 3 to 8-fold when expressed by plasmid. For R3A and JRFL Envs, surface expression was greater for TM1 Envs than for mutants lacking the +SIV segment or for the CT deletion mutant, ∆147. When 89.6 Envs containing the TM1 mutations, with or without the N_197_Q glycan deletion (N7) (Li et al., 2008), were expressed from a VACV vector previously used in poxvirus prime/protein boost vaccination protocols (Zarling et al., 1986, Cooney et al., 1993), an increase was also seen to levels 2–3 fold greater than WT Env (Fig. 3). In addition, 89.6 and 89.6 N7 Envs with TM1 mutations exhibited no change in reactivity with anti-Env mAbs, including several broadly neutralizing antibodies, indicating that Envs altered to enhance surface expression maintained epitopes in the Env ectodomain that are of interest to the vaccine field.

When the immunogenicity of 89.6 N7 Envs with WT or TM1-modified CT was compared using a VACV prime/gp120 boost protocol, the TM1 Env elicited significantly higher gp120-specific binding IgG and NAb to a Tier-1 HIV-1 isolate after two primes. Following the gp120 boosts, the difference in binding IgG was diminished, but the difference in NAb titers was markedly enhanced, up to about 16-fold. Strikingly, although mice primed with parental 89.6 N7 Env generated high levels of binding IgG, these mice exhibited poor NAb titers even after the second boost, indicating that significant qualitative differences in the antibody response resulted from CT modification. The lack of correlation between gp120-specific IgG and NAbs has been previously reported in multiple HIV-1 vaccine studies (Rerks-Ngarm et al., 2017, Buchbinder et al., 2017, Khattar et al., 2013), suggesting that monomer-specific antibodies are not necessarily neutralizing. The strain-dependent effects of TM1 modification on NAb activity and gp41-specific IgG are mechanistically unclear. It is possible that the Tier-1 NAb response elicited by JRFL TM1 was limited by the deficit in gp41-specific IgG in these mice compared to WT Env, or by a deficit in another epitope-specific response that was not measured. Future studies that focus on the effects of CT modification on the conformation and antigenicity of gp41 will likely be informative to this question.

An important finding in this study is that the improved NAb response after protein boosts in 89.6 N7 TM1-primed mice was attributable solely to differences in the VACV primes, which suggests the importance of the priming immunogen in determining the overall efficacy of this prime/boost regimen. This result is consistent with clinical studies of poxvirus prime/boost vaccines that have observed prime-dependent effects on antibody specificity, subclass, and neutralizing and non-neutralizing functions measured after the protein boosts (Chung et al., 2014, Graham et al., 1994, Yates et al., 2014). Although differences in immunogenicity were not as striking when JRFL Envs were used, these data suggest that, in some cases, high surface expression of Env could modulate the quality and the magnitude of antibody response and possibly improve efficacy of prime/boost vaccine protocols.

We hypothesize that, in our vaccine regimen, an increase in Env expression on the surface of professional antigen-presenting cells and potentially other cell types can enhance the ability of these cells to crosslink B cell receptors (BCR) and activate cognate B cells, resulting in increased Env-specific antibody responses. The multivalency of antigen/BCR crosslinking has a well documented effect on the efficiency of B cell activation, particularly for low affinity interactions (Bachmann et al., 1993, Ota et al., 2012, Jardine et al., 2013, Ingale et al., 2016). This strategy may preferentially elicit antibodies directed towards epitopes expressed on functional Env trimers presented by the prime that could be further improved by appropriate protein boosts. Indeed, it has been suggested that high-affinity binding to the functional Env trimer is both necessary and sufficient for an antibody to neutralize HIV-1 (Fouts et al., 1997, Tong et al., 2012, Parren et al., 1998). However, it is possible that the CT mutations we introduced could have contributed to immunogenicity through mechanisms other than surface expression. The SIV sequence in TM1 contains part of a motif that has been shown to activate NF-κB (Postler and Desrosiers, 2012), which can potently modulate host immune responses (Oeckinghaus and Ghosh, 2009, Yoshimura et al., 2001, Cancro, 2009). However, in preliminary experiments, we did not observe any difference in NF-κB signaling mediated by TM1 modification in the context of full-length Env or a CD8-Env CT fusion protein (data not shown) (Postler and Desrosiers, 2012). It is also well recognized that truncations in the HIV-1 Env CT can affect epitope exposure in the Env ectodomain, neutralization sensitivity, and fusion kinetics (Edwards et al., 2002, Chen et al., 2015, Wyma et al., 2004, Jiang and Aiken, 2007, Murakami et al., 2004, Joyner et al., 2011). Here, quaternary epitopes in the V1/V2 domain could not be probed using available broadly neutralizing antibodies (e.g. PG16, PGT145), as these do not bind to WT 89.6 or JRFL. Therefore, it is possible that antigenic differences beyond those tested here could have affected the immunogenicity of TM1 mutants.

Our findings build on previous studies that showed the ability of CT mutations to alter Env immunogenicity (Ye et al., 2004, Wyatt et al., 2008) and demonstrate that CT mutations designed to increase Env expression on the cell surface can enhance antibody responses in the context of a poxvirus prime/protein boost vaccine in mice. Although these findings will require validation in larger animal models, this result supports the idea that low surface expression of Env is an immune evasion strategy for HIV-1 and that the immunogenicity of Env-expressing vectors can be improved when Env is modified for high surface expression. An important aspect of these findings is that CT mutations can be combined with changes in the Env ectodomain that alter the quality or specificity of the antibody response (Li et al., 2008, Liang et al., 2016, Alam et al., 2013) and with improved boosting immunogens, such as stabilized SOSIP trimers (McCoy et al., 2016, Sanders et al., 2013, Sanders et al., 2015, Pugach et al., 2015), which have been shown to elicit NAbs to Tier-2 HIV-1 isolates (McCoy et al., 2016, Sanders et al., 2015). Further studies will determine whether CT modifications that increase Env surface expression have the same effect on immunogenicity in nonhuman primates and when delivered by alternative viral vectors or nucleic acid-based vaccines.
